# Book Reviews

**Published:** 1805-06-01

**Authors:** 


### CRITICAL ANALYSIS
### OF THE
### RECENT PUBLICATIONS
### ON THE
### DIFFERENT BRANCHES OF PHYSIC, SURGERY, AND MEDICAL PHILOSOPHY.

---

*A Treatise on febrile Diseases, including intermitting, remitting, and continued Fevers, eruptive Fevers, Inflammations, Hæmorrhages, and the Profluvia, in which an Attempt is made to present, at one View, whatever, in the present State of Medicine, it is requisite for the Physician to know, respecting the Symptoms, Causes, and Cure, of those Diseases: with experimental Essays, on certain febrile Symptoms; on the Nature of Inflammation; and on the Manner in which Opium and Tobacco act on the living animal Body.* By A. PHILLIPS WILSON, M. D. F. R. S. Edinb. Fellow of the Royal College of Physicians Edinburgh, &c. Vol. IV. 8vo. pp. 740. Winchester.

AT length, the fourth, and last volume, of this useful compilation has appeared; nor have we any reason to recommend it less to our readers, than any of the former. We wish, indeed, the printing had been more correct. For this deficiency, an apology is made, that the author was deprived of the opportunity of superintending the press. But, if a proper printer could not be procured in Worcester, 'tis pity a medical friend in Winchester could not have assisted in this necessary office. Nearly a page of two columns of errata is marked; and these make very few of the numberless errors; from which, our readers will scarcely believe, even that the title-page is not free.

This volume contains the fourth section of chap. ix. on *Cynanche Trachealis*, or Croup; x. *Pneumonia;* xi. *Peripneumonia* Notha; xii. *Carditis and Pericarditis;* xiii. *Gastritis;* xiv. *Enteritis.* xv. *Acute Hepatitis;* xvi. *Chronic Hepatitis;* xvii. *Splenitis*, *Nephritis*, *Cystitis*, and *Hysteritis;* xviii. *Rheumatism;* xix. *Gout.* Book ii. consists of *Hæmorrhage*, *Phthisis Pulmonalis;* of *Profluvia*, which is confined to dysentery, and finishes the work on febrile diseases. An appendix follows, as mentioned in the title.

Of the volume in general, suffice it to say, that it is got up with the same diligence as the former; contains the same extent of reading, the same display of authorities, and the same judicious remarks, on all that are quoted. If the author has been less successful in maintaining his own theories, than in detecting the errors errors of others, in this respect he only stands on the same ground as most of his brethren. As the work professes but little novelty, we shall select those articles which, on account of late controversies, are the most interesting.

In the first article, *Cynanche trachealis*, we have a very accurate account of all the symptoms, and a collection of almost all the authorities worth producing on the subject. The author has chosen to blend acute asthma with it, on account of the similarity of many of the symptoms and method of treatment in each. In considering the remote causes of both these diseases, indigestion is admitted, on the authority of Dr. Underwood, as one of the causes of croup; and Dr. Millar is said, "with great probability, to rank laxity of the solids, indigestible ingredients in the food, and a morbid weakness of the digestive organs, among the exciting causes of acute asthma. This kind of climax, or ringing the changes of laxity of solids, indigestion, &c. is, in our opinion, ill suited to the dignity of a work which undertakes to teach practitioners how to reason. The subjects of croup are more commonly stout, healthy children; and the inflammation is of that kind which, in a particular manner, marks strong vascular action. Laxity of solids, and indigestion, may be considered as causes of any thing, or of nothing; for the first is an expression of uncertain import, and the latter may be assumed whenever a practitioner thinks proper. Even where we find it an attendant on acute diseases, it is more commonly the effect than the cause. We have been the more careful to mark this, because it cannot but influence the practitioner in his mode of treatment. Dr. Wilson, with much propriety, urges evacuations, particularly free blood-letting, if the physician is called early enough to arrest the inflammatory symptoms. But, if we attend to the appearance on dissection, and the proximate causes, they must surely instruct us somewhat, in tracing the remote cause.

"On laying open the trachea of those who die of the croup, there is a membrane lining but scarcely adhering to it, for it may always be easily separated without destroying its shape. It comes out in the form of a tube, exactly adapted to the cavity it lay in. In many cases, indeed, it cannot be said to adhere at all; and there is a considerable quantity of puslike matter, lying between it and the the sides of the trachea. This membrane often extends beyond the division of the trachea, lining the large branches of the bronchiæ, and loosely adhering to them. The purulent matter extends beyond the membrane, often into the smallest branches of the bronchiæ, and even, in some instances, into the air vesicles. Mr. Wood found it in these vesicles, in the 7th and 8th cases, related by Dr. Home. On removing it, there is no appearance of ulceration in the coats of the trachea and bronchiæ; but the traces of inflammation are, in general, very obvious, and sometimes extend, Burserius observes, to the very extremities of the bronchiæ. It has sometimes happened, as in more than one dissection recorded by Dr. Home, that no traces of inflammation could be observed; but this is comparatively rare.

"By squeezing the lungs, a considerable quantity of a whitish, glutinous fluid may sometimes be forced out. In different cases of the cynanche trachealis, the lungs assume, according to Burserius, all the different appearances observed after pneumonia, which are soon to be laid before the reader. Sometimes, however, they are quite sound. The appearances of the lungs, in those who die of the croup, Michaelis observes, are various: sometimes they are sound; sometimes slightly inflamed; sometimes there is a sanious matter extravasated in different parts of them; sometimes the matter found in them is purulent, and sometimes merely a watery fluid, the quantity of which is often considerable.

"Small polypous concretions are often found in the vessels of the lungs, and in the right side of the heart; never, Burserius cbserves, in the left side, or in the aorta. These concretions, I have already had occasion to observe, seem to be formed in articulo mortis; and the reason of their not being found, in this case, in the left side of the heart, seems to be, that the blood is chiefly collected in the right, as happens in all cases where death is induced by suffocation. It is also owing to the dyspnœa which precedes death, that the vessels of the head are generally found very turgid in those who die of the croup.

"The preternatural membrane presents different appearances, in different cases. Sometimes, Michaelis observes, it is as thin as paper; in other cases, so thick, that it almost fills up the whole cavity of the trachea. It is often of different thickness, in different parts; and the thickest part is sometimes the uppermost, and sometimes the reverse. In some cases, it is soft and pulpy; in other cases, so firm and tough, that it will bear maceration in water for several days; but however tough it is in the trachea, it becomes more tender in the bronchiæ, and is always soft before its termination. In some cases, it is quite white; in others, marked with red spots; and it is now and then uniformly of a dark colour, and sometimes even black. Some have thought this membrane possessed a vascular, others a fibrous structure; the former opinion appears erroneous, and the latter is not confirmed by general observation."

On the proximate causes, we have the following remarks, from different writers, and our author's conclusions:

"It is necessary to say something of the different opinions which have prevailed, respecting the nature of this complaint.

"With regard to that which refers its seat to the lungs, and attributes it to an inflammation of this viscus, it will readily be admitted to deserve no attention.

"The first probable opinion, suggested on this subject, was that proposed by Dr. Home. He believed the first seat of the disease ease to be in the mucous glands of the trachea, which he supposes are excited to pour out an unusual quantity of mucus. 'When there happens,' he observes, 'a very great secretion of this coagulable fluid from the glands of the trachea in children, they are either not sufficiently attentive, or too young to spit it up. The thinner parts are carried off during expiration, while the remainder is thickened and compressed, by the obstruction which the narrowness of the glottis opposes to the exit of the air from a larger canal. Every circumstance,' he continues, 'encourages its concretion into a solid, firm membrane; while the more internal parts of the mucus continue still fluid, and the continual secretion of more keeps it separated from the parts below.'

"But Dr. Home explains the conversion of this mucus into pus, by experiments of Sir John Pringle; the inaccuracy of which I have already had occasion to notice. We have seen, besides, that the membrane may be formed without occasioning symptoms of croup; which, on the other hand, may exist without the formation of any membrane."

Dr. Rush's opinion follows; but this the author very properly considers as formed before Dr. R. had properly discriminated the disease. Michaelis considers the membrane as the cause of the disease; and that it is formed in the same manner as the polypi, found after death in the larger vessels. Our author very properly shows, that if the membrane is (like polypi) the effect of the coagulation of lymph, it must be formed in a different manner from those substances which can only occur by the coagulation of blood *in articulo mortis*.

Dr. Cullen's opinion next follows; which, like most of the physiological notions of that celebrated teacher, was connected with his favourite system of spasm. As, however, evident marks of inflammation appeared, and as antiphlogistic remedies were found necessary, he admitted this spasm to be connected with inflammation. But, says our author, who seems willing to concede as far as possible on this occasion, "what proofs have we of spasm on the glottis?" He afterwards adds, "Even inflammation is not always discoverable; but," says he, "it is far from being improbable, that the patient may die after the inflammation has ceased, the effusion being sufficient to occasion suffocation."

It is much to be regretted, that the eminent physiologist of London, whom all join in extolling, is so little read, or so little understood. The writers quoted by our author could not be well acquainted with those *fundamental principles of inflammation* which, by experiment, constant observations, and a mind formed to reason on the operations of nature, were established by Mr. Hunter. But all this was accessible to our author. It is, however, fair to make some allowance for the obscurity of language, of which Mr. Hunter was always accused, and which could not fail to be increased in a work published after his death. Yet a careful examination of part 2, chap. 4 (page 240 et seq.) would at once have solved solved all the difficulties that Dr. Wilson met with, in reconciling the contradictory inferences drawn by his various authorities from the different facts admitted by them all. In these passages, and the others pointed out therein by Mr. Hunter, it will appear, that the different parts of the body are divided, by him, according to the nature of their surfaces, and their consequent capacity to take on different kinds of inflammation, into two orders. The first order consists of the cellular membrane and circumscribed cavities: the second, all the outlets of the body, commonly called mucous membranes; for instance, all the ducts of the glands and the alimentary canal.

The first order takes on the adhesive inflammation, in the first instance, preparatory to suppuration and ulceration. The adhesive inflammation consists of effusion of lymph; the coagulation of which, by uniting the parts together, serves to prevent the spreading of the inflammation, and, in case of future suppuration, to confine the matter from diffusing itself through the cellular membrane or loosely into the cavities. By these means, the disease is as circumscribed as possible, and the functions of the parts sustain the least injury. But, in secreting surfaces, the case is quite different. Should the adhesive inflammation take place, the function of the parts must be for ever destroyed, and the injury to the economy would be in proportion to the office of the part. Inflammation in these parts, therefore, runs immediately into suppuration, by an alteration of the secretion, without any previous effusion of coagulating lymph. There are instances, however, in which so violent an inflammation may be excited on these surfaces, as to induce a process similar in part to the adhesive inflammation; that is, instead of the suppurative process, or the change of the secretion from mucus to pus, coagulable lymph may be thrown out. But, in this case, it will not produce that general adhesion which takes place in the first order of parts. On the contrary, the lymph will either be loose in the canal, or adhering only at certain points; and, as soon as the first violence of the inflammation ceases, suppuration will commence, with an attempt to dislodge the effused coagula, by absorbing the adhering points, and by ejecting the rest by the outlet peculiar to the part. Mr. Hunter enumerates the various canals and surfaces in which he has seen this process. Among the rest, he mentions the trachea; and, to demonstrate that the difference in the effect of inflammation, in such parts, may arise from its greater violence, he produced the effect artificially, by injecting a strong solution of corrosive sublimate up the vagina of an ass. But that eminent physiologist was much too cautious to assert, because, in this instance, the effusion of coagula arose from the greater violence of the inflammation, that therefore no other cause would produce such an effect on secreting surfaces. On the contrary, he suggests, that, in some cases, the inflammation is of the erysipelatous kind; so that, should any other cause produce erysipelatous inflammation on secreting surfaces, the same effect might follow, though the inflammation should not be particularly violent.

From these premises we may readily account for the prevalence of croup, at particular periods; as we well know, that certain constitutions of the air are productive of erysipelatous inflammation. The nature of the membrane, its consequent disappearance, and the purulent surface of the trachea, are also easily accounted for. After the first effects of the erysipelatous inflammation has ceased, if the disease is not particularly severe, the parts return to their common offices. Suppuration takes place as the effect of altered secretion, on such surfaces, and also to dislodge the parts of coagulum which adhere. Part of the coagulum is absorbed; the remainder is thrown up in different portions, sometimes so small as to escape detection, and to be swallowed by the young patient.

This knowledge of the ætiology of the disease would very much have assisted our author, in reconciling the different modes of treatment proposed by different physicians. We well know, that the endemic, at different periods, assumes different degrees of action, though the general form is the same, and, as he observes, that the different periods of the same disease require different modes of treatment. On this subject, it is but justice to add, that our author is very full and perspicuous. Perhaps he may show more caution, as to copious blood-letting, than is necessary. In the early stage of the disease, it must be continued as long as symptoms of high inflammation remain, for so long will the danger of an increased effusion of coagulum continue. Emetics also are highly serviceable, in producing a new set of actions, and on a new part of the system. However, it is unnecessary for us to dilate on this part of the subject, not only because Dr. Wilson has left little to add, but because, in diseases so violent, if the practitioner has not courage to assume a discretionary power, it will be in vain to instruct him.

We have been the more particular on this disease, because of the urgency of all its symptoms, and because, in our opinion, the causes and progress have not hitherto been marked with sufficient accuracy.

In the next chapter, on pneumonia, Dr. Wilson is particularly full. The frequent occurrence of the disease rendered this the more necessary. Throughout the whole, our author shows his former industry and perspicuity. Nor have we less to commend in his attempt at a proper distinction between this disease and the peripneumonia notha. Gastritis succeeds, and the remarks are, for the most part, equally unexceptionable. Acute and chronic hæpatitis, with inflammation of the other viscera, according to their names, follow in order; and the mode of treatment being tolerably uniform, the author very properly dismisses the latter with a few general remarks.

The subject of rheumatism our author introduces by some very judicious remarks on the modern attempts at nosology. We have often regretted the number of hard Greek derivatives which boys, ignorant of the characters of that language, are taught at one of the the British universities. These technical phrases are the choicest food for rearing coxcombs, whilst they prove the greatest incumbrance to the ingenuous student. But on this subject we shall leave our author to speak, who certainly would not, without reason, calumniate his *alma mater*.

"It is with little propriety, that either the chronic hepatitis or chronic rheumatism are arranged under the definition of phlegmasiæ. Dr. Cullen could not, however, have arranged them otherwise, without having made a material alteration in his system. Besides, the immediate connection of these with the acute forms of the disease, seems to afford an apology for the place which they hold in his nosology.

"The only method of forming an accurate system of nosology is, to class diseases according to their symptoms; and where we find the symptoms so dissimilar, and the nature of the complaints so much allied, we are at a loss how to proceed.

"Whatever is done in a system of practice, it would seem that in a system of nosology we ought to study the symptoms of diseases alone. If any other principle is admitted, the most dissimilar affections will frequently be classed together, according as the hypotheses of different writers lead them to conceive diseases are allied to each other. There is no better reason for arranging the acute and chronic rheumatism or hepatitis under the same order of diseases, than for arranging together any other complaints which insensibly run into each other.

"It would seem, that in forming a system of nosology, our study should be to select the symptoms which characterise each disease in its perfect form, and arrange those together whose symptoms are most similar, without attending to the various gradations by which diseases run into each other. Is it our aim, in forming such a system, to assist the learner, in enabling him to distinguish diseases? This is the simplest way. After he is acquainted with such a system, a single perusal of a system of practice will teach him the connection which diseases have with each other. This is not the purpose of nosology. Is it our aim, in forming such a system, to assist the practitioner? This is the way in which we shall be most successful. I have frequently had occasion to observe, that where the symptoms are similar, the modes of treatment generally are so likewise. Even according to this plan, indeed, we shall often class together very different diseases, because in their most striking features they agree. Synocha and typhus are classed together, notwithstanding they are very dissimilar in their mode of treatment. But by classing diseases merely according to their symptoms, we shall, I believe, class together a greater number of diseases whose treatment is similar than by pursuing any other method.

"To see into what errors our best nosologist was led, by losing sight of this principle, it is only necessary to peruse Dr. Cullen's order of Spasmi. It looks like a common receptacle, for the refuse of of the whole nosology. This order is ready to receive every disease that is rejected by the others. Where is the similarity between hydrophobia and diabetes, between cholic and hooping cough, between dyspnœa and epilepsy, &c.? It may be affirmed, I believe, that there is no person, unacquainted with Dr. Cullen's peculiar opinions, who can point out for what reason these complaints have been classed together. If he was at a loss where to arrange any disease, it was only necessary for him to call it a spasm, and it found a place in one of the chief orders of his nosology.

"No attention to symptoms of diseases, perhaps, will enable us to form a perfect system of nosology. The difficulties are far greater than at first sight they appear to be; but there is reason to believe, that, by steadily pursuing this plan, we may approach more nearly to such a system than has hitherto been done. If this plan is confessedly the best, ought we not to proceed on it as far as we can; and when we meet with difficulties which cannot be overcome, point them out, and leave them to be removed by future observation, rather than give our systems the appearance of being complete at the expence of truth, or of proceeding on a principle which can be of little or no use either to the student or the practitioner. However ingenious, nay, however just a theory may be, it should have no place in a system of nosology, which is a mere classification of terms, whose sole objects are to give accurate definitions of the names of diseases, and class together those definitions which most resemble each other."

Our author proceeds to shew, that Dr. Cullen's definition of Rheumatism is defective. He then enters on a description of the symptoms, and follows the subject according to the order he has pursued in the rest of the work. The reader will find many useful remarks; and we particularly approve the cautions against a too long continued use of the lancet. If early copious bleedings do not succeed, the disease must be considered as chronic, and treated as such

Under the article gout, we are glad to find our author not only copy Sydenham's *picture*, but also acknowledge the obligations he owes. This he very properly accuses most writers, and, among the rest, Dr. Cullen, of neglecting. It is strange, that on subjects of philosophy men should not be anxious to strengthen themselves by such an authority, or that they conceive it possible their plagiarism would be overlooked when the sources are not only open to, but explored by every one.

Dr. Wilson is very careful to follow Sydenham throughout; and whilst he boldly asserts that gout is not to be ranked with common phlegmasiæ, instead of pointing out the difference, he satisfies himself with proving, from the authority of his guide, that bleeding is rarely to be admitted. At the beginning of the work a caution is prefixed, lest any thing here said should seem to reflect on Dr. Kinglake, whom our author asserts he had not read when his remarks were written. There can be no doubt of this, as no

 mention mention is made of the exhibition of cold water in this disease; a controversy we are the more surprised to see overlooked, as the practice is much older than the present generation. The whole of this section is fuller of caution than information. Whatever has been observed by others is faithfully recorded, excepting indeed, that the great pattern proposed for our imitation is not sufficiently followed through his manner of treating himself. Under the suspicion of a stone in the kidneys, Sydenham informs us, it was his custom to take a purge once a week, and this he did without any greater recurrence of gout, than what was easily obviated by an opiate on the evening after taking his purge. It is not our business to reconcile the discordant opinions which have ever been promulgated concerning this disease; and we are ready to give our author credit for the impartiality with which he produces authorities from all quarters. But we wish he had done this, reserving his remarks till the conclusion. His own opinion might then have been fairly canvassed. Instead of this, we have, at the close, or in the midst of each writer's practice, a set of bye remarks, which show the author is averse from all bold remedies. Cold, being the most successful of all, he considers as the most dangerous; and concludes with admitting flannel and well-combed wool as the safest topical remedies, because, if they do no considerable service, they at least do no harm. We shall conclude our remarks on this chapter, by wishing that our author had consulted Dr. Hebberden's commentaries before he finished this part of his work.

The chapter on hæmorrhage affords nothing new. That on phthisis pulmonalis is very full, but chiefly of the observations of other writers.

Under the article profluvia, we have an ample description of dysentery, with the opinions of a variety of writers, and some good remarks of our author. We must, however, except his extreme caution on the subject of blood-letting. For common practitioners these cautions may be of little consequence, as the disease is rarely serious in these temperate regions, as long as men live in what may be a natural state of society. But in the warmer climates, in camps, or in that unnatural state with which the military life is attended, early and free bleeding is often necessary, to arrest the violence with which every disease assails a constitution under such circumstances. A great deal of the contrariety of opinion remarked by Dr. Wilson might have appeared less striking, if he had considered that he was quoting two distinct classes of writers, the military and civil physicians.

Among the cathartic remedies, our author considers ipecacuanha, in small doses, as the most deservedly celebrated. He is, however, much more sparing, in producing his authorities for this, than in favour of other remedies. In his own practice, he discovers something more than a mere cathartic quality in ipecacuanha, viz. "the relaxation it produces on the skin, which is always accompanied with a tendency to similar relaxation in the alimentary canal."

canal." That is, if we understand him, that the great increased action of the intestines is diminished; and for this purpose the remedy was usually exhibited, till Dr. Cullen gave it as his opinion, that it had no virtues but that of purging. This is, however, by no means certain. The cathartics produce their effect by restoring, in some measure, the natural action of the peristaltic motion, and by lessening the tendency to inflammation. Ipecacuanha, by its nauseating property, lessens the high irritation and consequent unnatural action of the canal. For these reasons, the latter is found a very feeble remedy in camp dysenteries, though highly serviceable in common practice

An appendix is added to this volume, containing three essays: two of them experimental; one on the manner in which opium acts on the living animal body, the other containing similar experiments on tobacco. In the first, the author examines, with much candour, what has been done by his predecessors, and afterwards adds his own experiments. The result of the whole is, that opium produces no effect but on the parts to which it is substantially applied, either by immediate apposition, or by the absorbents and blood-vessels; that is, that when it affects the head it must be injected on the brain, or carried thither by the blood-vessels: that if it affects the heart, when injected into the abdomen, it arises from the extreme vessels being paralyzed, and the blood, in consequence, accumulated in the heart; that the nerves are only affected as they come in contact with the opiate solution itself, and not by sympathy.

In the experiments on tobacco, similar results followed.

The third essay is on the doctrine of sympathy of nerves. This abounds with close reasoning on well-established facts. Without doubt, the sources of sympathy are various, seemingly dependent on absorbents and other vessels, as well as nerves, and, in many cases, we are apt to confound associations with sympathy.

Such are the contents of this useful volume, which we heartily recommend to the young practitioner. Impartiality, however, obliges us to dismiss the article with two remarks. The first is, that, in most acute cases, we discover too much caution in the treatment of early symptoms, without impressing the young practitioner sufficiently with the importance of decisive measures during that period of the disease. The second is, that, though, by the number of quotations from all quarters, it is evident the whole has been compiled with much diligence, yet the inattention to modern authorities makes us suspect the work has been concluded several years ago, and, in the revision, that the author, probably on account of professional engagements, has not sufficiently attended to contemporary writers.

---

*A General Treatise on Cattle, the Ox, the Sheep, and the Swine, comprehending their Breeding, Management, Improvement, and Diseases; dedicated to the Right Honourable Lord Somerville.* By JOHN LAWRENCE, Author of the New Farmer's Calendar, Modern Land Steward, &c. &c. Octavo, pp. 635. London.

THIS appears to us, a good practical book. We pretend not to be competent judges of farming; but we have been much struck with the medical department, which is divested of the nonsensical physiology of pretenders to a knowledge of the diseases of cattle. The descriptions of some of the maladies are too short, but they are not likely to mislead any one by the obscurity of the language, nor to puzzle a clown or make a philosopher smile by the wildness of the theories. The proposed remedies are also simple and rational, and we hope, in the next edition, the work will be extended to two volumes, and this department proportionally enlarged.

---

*A Botanical Dictionary, or Elements of Systematic and Philosophic Botany.* By COLIN MILNE, LL. D. Author of Institutes of Botany and Habitations of English Plants. Third edition, revised, corrected, and very considerably enlarged, illustrated by twenty-five plates. Octavo. London, 1805.

THIS work contains a great deal of Botanical information, conveniently disposed and accurately compiled. The plates are well executed, and very numerous, the style simple and clear, the descriptions accurate, the remarks well founded, and the arrangement as compleat as the nature of the performance admits. We think, however, this edition might have been enlarged in some parts without injury to the purchaser.

Under the article "Sexes of Plants," we were surprized to see no notice taken of Spalanzani's numerous experiments. Mr. Knight's experiments too, of impregnations with different pollen, should have been at least hinted at. Whether all the advantages the inventor promises have been experienced by others, we have been unable to learn.

With all these and other smaller omissions, we congratulate our readers on the appearance of this edition, and recommend it to their perusal.

---

### *Dr. Jackson, on the Treatment of Fevers.*

( Continued from pp472.—475. )

When due preparation has been made by unloading the sanguiferous system, when necessary, and clearing the primæ viæ, Dr. J. proceeds to his grand remedy, BATHING. In his hands this appears to be almost a specific. Dr. Currie had informed the public, in England at least, of its great utility in the cure of fever, when certain certain conditions are present, and of its danger when other conditions are present; but Dr. J. has taught us how to produce by art those requisite conditions, by which means the utility of bathing is rendered almost universal. Although this veteran in febrile practice has much improved every part of the treatment of these formidable diseases, yet we consider his mode of applying water as by far the greatest improvement that he or any other practitioner ever made. Impressed with this conviction, our readers will naturally expect a pretty full detail of his instructions and discriminations on this subject. We shall not disappoint them.

" When the perverted motions which obtain in fevers, or rather, in which fevers consist, have been arrested by the effects of bleeding, emetics, or other means; or, though the motions be not actually arrested, when susceptibility of impression has been restored, by these and other suitable processes; the next, the great and the final effort, is directed to the means of bringing back a movement, similar to that of health. When the impression of the cause of diseased motions has been removed; or when the condition, under which it acts, has been changed, or weakened, the application of the common stimulants of life is often sufficient to recall the natural movements of the machine. Where this is not the case, and there are many instances where it is not, the attainment of the effect is committed to other powers. Of these, bathing, that is, the application of water to the surface of the body, alternately warm and cold, has a powerful operation; and it is considered, with justice, as a most powerful means of originating new motions. This remedy has been known since the time of the Emperor Augustus; and its use and management were well known to Galen, and well defined by him. It farther appears, by the relations of travellers, to have been, and to be even now, a practice with several of the Eastern nations. It was tried by Dehahn at Warsaw, in the year 1737; and rubbing of the body with snow, a process somewhat similar, but still more effectual than cold bathing, was employed by Samoilowitz in the plague of the year 1771. These facts existed, but they seemed not to have made any impression; for bathing in fevers, has not been reckoned among the regular means of regular physicians, till very lately. It has now attracted some notice in England,— a notice chiefly due to the popular manner in which the subject has been treated by Dr. Currie, of Liverpool; for though the remedy has been employed by the author for near thirty years, that is, since the year 1774; and though the knowledge of it has been before the public since the year 1791; as well as that the fact had been communicated to several persons before that time; it is not probable that it made much progress, in consequence of his recommendation.

" In the year 1774, cold bathing was not a remedy commonly recommended by medical teachers, or medical writers, in the cure of fever. The hint, here acted upon, first suggested itself from the relation of a fact mentioned by a seafaring man, who had been master of a transport ship at the siege of Havannah. In noticing cursorily, among the events of that service, that several of the men, who were on board of his ship (which was a kind of hospital ship), threw themselves into the sea in the delirium of fever, that some were drowned, that others were recovered from the waves; it was asked, if he recollected what was the effect upon health. He had noticed the fact, and remembered the event:—the delirium ceased, and the greater number recovered. The fact, which was candidly expressed, made a strong impression.' An opinion was suggested by it, and that suggestion was strengthed by an event which occurred in the island of Jamaica, early in the year 1774. A negroe child was lying in a piazza, apparently within a few minutes of death, in the secondary fever of small-pox. A pail of water was by chance at hand, the presence of which probably connected the ship-master's relation with the present case. The negress who attended the child was desired to sprinkle its face and breast. It was done;—the effect was striking; for the apparently dying object was instantly revived. The effect, however, was only temporary; the former state recurred again; the affusion was repeated, and the effect was similar.—By repeating this process at intervals, life was preserved for upwards of twelve hours, when apparently it could not have gone on, without such means, for as many minutes. The effect was singular,—similar to what follows the affusion of cold water upon a dying fish, the subject of the experiment reviving and sinking alternately, according as the means were employed or withheld.

"With the relation of what happened at the Havannah in mind, and the example of what happened to the negroe child before the eye, the subject of cold bathing strongly attracted the author's attention. The practice, in short, was adopted by him in the year 1774. It was tried without fear; and the effects were favourable beyond expectation. It was employed freely in the West Indies: and it was even tried in England, prior to the year 1791, though only in a few instances; for the prejudices against such a remedy were not so easily to be overcome. The experiments of the practice have, however, been so numerous, and the proofs of the benefits so multiplied, so varied, and so amply extended in different climates, and in different conditions of disease, during the late war, that the memory of what happened in the preceding period may be allowed to be superseded, or blotted out. Since the year 1791, that the author's treatise on the fevers of Jamaica made its appearance, his experience has been exercised in an ample field; and his opportunities of witnessing the effects of bathing, have probably been greater than those of any other person in Britain, probably than any other person in Europe. Without predilection in favour of a remedy, of which, though not the discoverer, (for the discoverers are not of this age or country,) he he assumes some claim in defining the principle which ought to direct the application; he does not hesitate to say, that, if there be a charm among the means employed for the relief, or abrupt cure of fever, it is found in a judicious management of warm and cold bathing. The proofs are not confined to solitary instances; for it has occurred almost daily, that persons who had entered the bathing room, under symptoms of the most threatening aspect, have demanded their clothes, that they might return to their military duties, after the routine of operation performed in this place was completed, — a routine, of which bathing was the last and most important part. In fevers of a certain form, in a certain stage of progress, or after a certain preparation, the effect is generally decisive of health.

"But in order to be able, in all cases, to employ so powerful a remedy with advantage, it is previously necessary to investigate minutely, and establish demonstratively, the principle upon which it acts fundamentally, as well as to define correctly, the circumstances, which favour or oppose its successful operation. Bathing, like every other power in nature, acts upon the excitability of organism, and produces, more obviously than most others, an effect upon organic movement. It is immediately applied to the surface of the body, it consequently acts upon the surface, and produces effects, corresponding with its own power, and the capacities of the part upon which it acts. From attending minutely to the circumstances of the conditions, in which it appears to produce the greatest change or greatest good; susceptibility of impression, with a power of producing action, termed excitability, seems to be that alone, which is uniformly and radically connected with its operation. According to the degree of this condition may be estimated, *à priori*, the effect of the means. The means are in this case applied to the surface; and as the change produced, is in proportion to the power of the cause, and the condition of excitability of the part, to which the cause is applied; so the effects are proportionally most remarkable, where the surface is most excited,—or most excitable. The balance is then ticklish, and a new train of action originates from a slighter cause.

[To be continued.]

